# The Need for an Individual Approach to Lung Cancer Treatment

**DOI:** 10.1371/journal.pmed.0030206

**Published:** 2006-04-25

**Authors:** Hisayuki Shigematsu, Shinichi Toyooka, Makoto Suzuki

**Affiliations:** **1**Okayama University Graduate School of Medicine and DentistryOkayamaJapan; **2**Okayama University Graduate School of Medicine and DentistryOkayamaJapan; **3**Chiba University Graduate School of MedicineChibaJapan

Heidi Greulich and colleagues [ 1] have demonstrated the significance of insertion mutations in the epidermal growth factor receptor (EGFR) (exon 20) for tumorigenesis and responsiveness to tyrosine kinase inhibitors (TKIs) in lung cancers. They reported that different types of *EGFR* mutations conveyed different sensitivity to TKIs. Although several previous studies have suggested that tumors harboring mutations in the EGFR kinase domain were sensitive to TKIs such as gefitinib or erlotinib [ 2, 3], others had reported an association between the *T790M* mutation in exon 20 and the resistance to TKIs [ 4, 5]. As we learn more about the relationship between *EGFR* status (including gene copy number, mutation status, and mutation type) and drug sensitivity, decisions about treatment with TKIs for patients with non-small-cell lung cancer (NSCLC) become more complex.

Previously, we reported *HER2* mutational status, as well as *EGFR* and *KRAS*, in a large number of NSCLCs [ 6]. We found that 22% of tumors had *EGFR* kinase domain mutations (149 out of 671). Of these 149, 15 of them were insertion mutations in exon 20. The more common types of mutations—i.e., deletion in exon 19 (68 out of 149) and L858R in exon 21 (61 out of 149)—were more frequent in women and in “never smokers” (with *p* values of less than 0.001), whereas the exon 20 insertion mutations showed no bias for sex (seven in males versus eight in females) or smoking status (seven in smokers versus eight in never smokers). We have no data regarding TKI sensitivity for the 15 patients with insertion mutations to date, but based on the results from others and our own in vitro data, they may not benefit from the conventional TKIs, despite the fact that their tumors have *EGFR* kinase domain mutations.

To maximize benefit to patients, we should determine the exact type of mutation for an individual tumor and determine whether it conveys sensitivity or resistance prior to TKI therapy. The development and clinical application of novel agents overcoming resistance should yield a more effective targeted therapy for tumors with all types of *EGFR* mutations.

## References

[pmed-0030206-b1] Greulich H, Chen TH, Feng W, Janne PA, Alvarez JV (2005). Oncogenic transformation by inhibitor-sensitive and -resistant EGFR mutants. PLoS Med.

[pmed-0030206-b2] Paez JG, Janne PA, Lee JC, Tracy S, Greulich H (2004). EGFR mutations in lung cancer: Correlation with clinical response to gefitinib therapy. Science.

[pmed-0030206-b3] Lynch TJ, Bell DW, Sordella R, Gurubhagavatula S, Okimoto RA (2004). Activating mutations in the epidermal growth factor receptor underlying responsiveness of non-small-cell lung cancer to gefitinib. N Engl J Med.

[pmed-0030206-b4] Kobayashi S, Boggon TJ, Dayaram T, Janne PA, Kocher O (2005). EGFR mutation and resistance of non-small-cell lung cancer to gefitinib. N Engl J Med.

[pmed-0030206-b5] Pao W, Miller VA, Politi KA, Riely GJ, Somwar R (2005). Acquired resistance of lung adenocarcinomas to gefitinib or erlotinib is associated with a second mutation in the EGFR kinase domain. PLoS Med.

[pmed-0030206-b6] Shigematsu H, Takahashi T, Nomura M, Majmudar K, Suzuki M (2005). Somatic mutations of the HER2 kinase domain in lung adenocarcinoma. Cancer Res.

